# Evaluation of the role of clathrin and bacterial viability in the endocytosis of *Lawsonia intracellularis*

**DOI:** 10.3389/fvets.2023.1005676

**Published:** 2023-01-30

**Authors:** Carlos Eduardo Real Pereira, Talita Pilar Resende, Amanda Gabrielle de Souza Daniel, Fabio Augusto Vannucci, Connie Gebhart, Roberto Mauricio Carvalho Guedes

**Affiliations:** ^1^Department of Clinic and Surgery, Escola de Veterinária, Universidade Federal de Minas Gerais, Belo Horizonte, Minas Gerais, Brazil; ^2^Department of Veterinary and Biomedical Sciences, College of Veterinary Medicine, University of Minnesota, St. Paul, MN, United States

**Keywords:** endocytosis, intracellular bacteria, infection, pathogenesis, proliferative enteropathy

## Abstract

*Lawsonia intracellularis* is an obligate intracellular bacterium and causative agent of proliferative enteropathy. The pathogenesis of *L. intracellularis* is not completely understood, including the endocytic mechanisms to access the host cell cytoplasm. In this study, we evaluated the mechanisms involved in endocytosis of *L. intracellularis in vitro* using intestinal porcine epithelial cells (IPEC-J2). Confocal microscopy was used to co-localize *L. intracellularis* and clathrin. Clathrin gene knockdown was then applied to verify whether *L. intracellularis* endocytosis is clathrin-dependent. Finally, internalization of viable and non-viable (bacteria were inactivated by heat) *L. intracellularis* organisms were assessed to study the role of the host cell during bacterial endocytosis. *L. intracellularis* organisms were observed co-localized with clathrin by confocal microscopy but the amount of *L. intracellularis* internalized in cells, with and without clathrin knockdown, did not differ statistically. The internalization of non-viable *L. intracellularis* showed a decrease in the internalization in cells with less clathrin synthesis (P<0.05). The present study is the first to elucidate the involvement of clathrin in the endocytosis of *L. intracellularis*. Clathrin-mediated endocytosis was shown to be an important, but not required, process for *L. intracellularis* internalization in porcine intestinal epithelial cells. Independence of bacterial viability for host cell internalization was also confirmed.

## 1. Introduction

*Lawsonia intracellularis* is an obligate intracellular, microaerophilic, Gram-negative bacterium that causes proliferative enteropathy (PE) (1). This disease is endemic in swine herds worldwide, and is also reported in other species, including non-human primates, horses, rabbits, wild mammals, and birds (2, 3).

The pathogenesis of *L. intracellularis* is still poorly understood. *L. intracellularis* infection occurs *via* the fecal-oral route. In the gastrointestinal tract, the microorganism overcomes the hostile environment of the stomach and localizes in the aboral region of the small intestine (ileum) (4). The bacterium uses a single polar flagellum (5) to cross the intestinal mucus barrier, allowing intimate contact with the enterocytes and subsequent host cell invasion.

Endocytosis of intracellular bacteria can occur *via* different routes. The zipper mechanism does not require metabolically active invaders for its initiation. The zipper endocytosis process is associated with proteins or structures of the outer membrane of the bacterium that interact directly with specific host cells receptors. In this process, there is a clustering of eukaryotic cell receptors, associated with changes in the actin cytoskeleton resulting in the microorganism being enwrapped by cytoplasmic membrane (6, 7). Then, several downstream mechanisms are activated resulting in pathogen endocytosis. In the zipper endocytosis process, there is involvement of clathrin-dependent machinery, associated with the formation of vesicles from the endocytosis of exogenous material. The involvement of this machinery occurs in the initial stage of the endocytosis process, shortly after the adhesion of the bacterium to the surface of the host cell (8). Another method used by bacteria for internalization into cells is the so-called trigger endocytosis mechanism. This process is dependent on bacterial effector proteins that are introduced into the host cells, generally through the type III secretion system (T3SS) (9). These proteins are responsible for mobilizing the actin for rearrangement of the cytoskeleton and pathogen internalization (9).

The mechanisms involved in the endocytosis of *L. intracellularis* are not yet fully understood. Lawson et al. (10) have demonstrated that *L. intracellularis* can be internalized even after its inactivation. This finding indicates that the process of *L. intracellularis* internalization in the host cells is dependent on the recognition of bacterial membrane proteins by the host, and hence, the clathrin-machinery would be potentially involved. Therefore, the objective of the present study was to evaluate the involvement of clathrin in the endocytosis process of viable and non-viable *L. intracellularis*, as well as to determine whether the bacterium uses other mechanisms for its internalization in the host cell.

## 2. Results

### 2.1. *L. intracellularis* and clathrin were co-localizated by confocal microscopy

**Figure 1:**
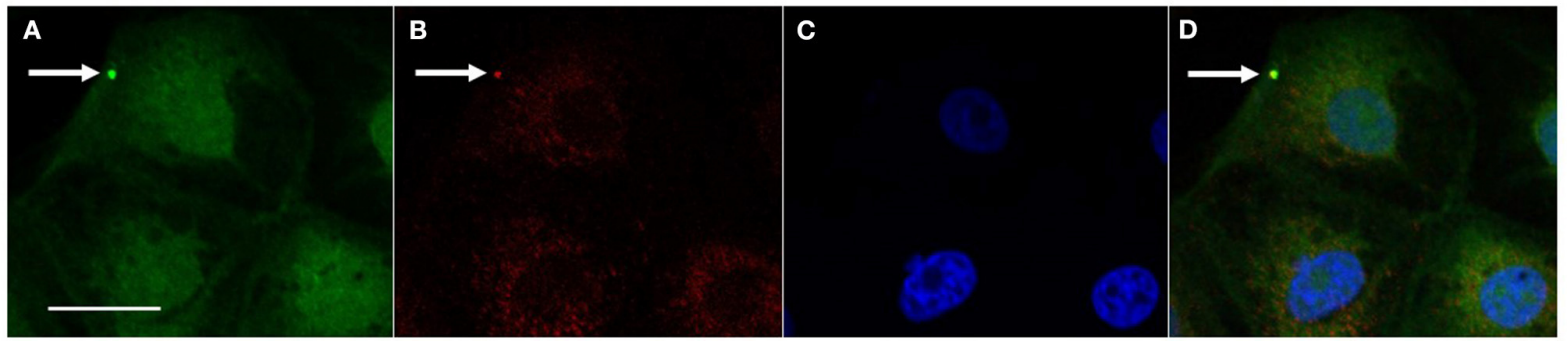
Co-localization of *L. intracellularis* and clathrin in IPEC-J2 cells 1 h after inoculation. **(A)** *L. intracellularis* fluorescent positive signal (arrow, green signal, AlexaFluor 488) and **(B)** Clathrin fluorescent positive signal (arrow, red signal, Cy3) were observed in the cell cytoplasm. **(C)** Cell nuclei (blue—DAPI) and **(D)** Co-localization of the fluorescent signals for *L. intracellularis* and clathrin (arrow, yellow signal). Confocal microscopy„ scale bar: 10 μm.

The co-localization of *L. intracellularis* and clathrin was observed by confocal microscopy and indicates an activation of the clathrin-dependent endocytosis mechanism to internalize *L. intracellularis* (Figure 1).

### 2.2. Internalization of *L. intracellularis* is affected by bacterial viability and clathrin expression

**Figure 2:**
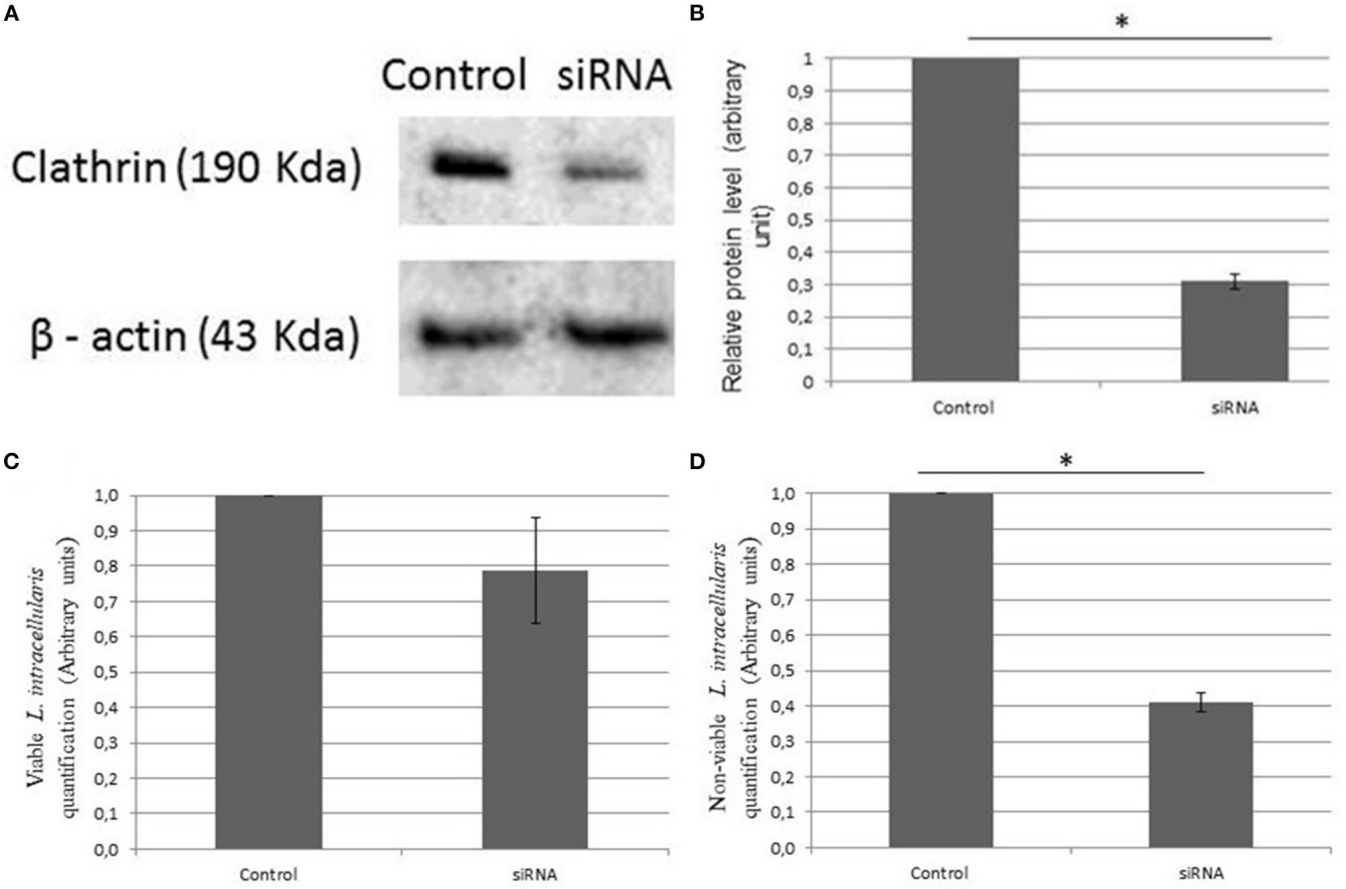
**(A)** Effect of clathrin knockdown using Western blot. The expression of clathrin in culture of IPEC-J2 infected with *L. intracellularis* demonstrated a lower expression rate of clathrin in the transfected group (siRNA). β-actin was used as loading control. **(B)** The relative protein levels analyzed by ImageJ and standard deviation. **(C, D)** The evaluation of the amount of *L. intracellularis* endocytosis in the different groups was performed using RT-PCR. Results are presented as the means ± SD of three independent experiments, using control groups (IPEC—J2 infected with *L. intracellularis*) and siRNA groups (IPEC—J2 knockdown to clathrin and infected with *L. intracellularis*). **(C)** Live *L. intracellularis* amounts did not differ statistically in the control and transfected group and **(D)** heat killed *L. intracellularis* had a reduced internalization rate (*P < 0.05) in the transfected group when compared to the control.

Clathrin knockdown by siRNA transfection was successfully demonstrated by the western blot technique (Figure 2A), and relative protein levels were reduced by 68.9 ± 2% (Figure 2B). Quantification of bacterial internalization was evaluated by cell culture lysis (control and siRNA) and bacterial extraction and quantification through RT-PCR. We observed that viable *L. intracellularis* had a numerically higher internalization capacity than heat-killed *L. intracellularis* (but not statistically, *P* > 0.05) in cells able to synthesize normal levels of clathrin (Figure 2C). Conversely, internalization of heat-killed *L. intracellularis* was significantly decreased (*P* < 0.05) in clathrin knockdown IPEC-J2 cells (Figure 2D), compared to non-treated cells.

## 3. Discussion

*L. intracellularis* is a relatively “new” bacterium species with the first report of isolation and *in vitro* propagation dating from 1993 (1), and its recognition as a genus and species in 1995 (11). *L. intracellularis* is the single species in its genus and shares only 92% of identity with *Bilophila wadsworthia*, the closest bacterium species in the same family. In addition, although available in public databases, *L. intracellularis* genome still lacks characterization of various sequences. The reproduction of pathogenesis published studies performed with other enteropathogens such as in *Salmonella enterica, Listeria monocytogenes* and *Escherichia coli* is very difficult due to growth particularities of *L. intracellularis*. The lack of understanding about the molecular biology of *L. intracellularis*, associated with the microaerophilic and intracellular requirements for *L. intracellularis in vitro* growth, make *L. intracellularis* a unique pathogen and dictate the realization of proof-of-concept experiments before any deeper and more comprehensive experiments can be performed.

In the present study, internalization of heat-killed *L. intracellularis* was significantly lower (*P* < 0.05) when infecting IPEC-J2 cells under a clathrin gene silencing condition. The method of bacterial inactivation used in the present study (65°C/30 min) would have likely minimal impact on surface structure (12), and hence, would likely not interfere on the recognition of membrane proteins as it was evidenced by the success of the internalization of heat-killed *Lawsonia intracellularis* in IPEC-J2 cells with full expression of clathrin. Altogether our results showed evidence that *L. intracellularis* can be internalized by clathrin-dependent mechanisms without losing the property of being endocytosed by other alternative active pathway processes. In other words, based in our results it is possible that viable *L. intracellularis* could also be internalized by a mechanism that does not involve clathrin pathway. Further studies are needed to evaluate the intracellular traffic of *L. intracellularis* within epithelial cells.

The importance of *L. intracellularis* as a pathogen capable of causing disease of economic importance in the swine industry is well-acknowledged. Nevertheless, little information on the pathogenesis and, more specifically, on the mechanisms of entry of the bacterium into eukaryotic cells, is available. In a previous study, Lawson et al. (10) have observed that dead *L. intracellularis* could be visualized inside cells, suggesting that the process of endocytosis could be dependent on the eukaryotic cell only. In the present study, we evaluated the mechanisms involved in the endocytosis of *L. intracellularis* and confirmed, by confocal microscopy, that *L. intracellularis* can be internalized through clathrin-dependent mechanisms. However, the amount of live *L. intracellularis* organisms recovered from transfected and non-transfected IPEC-J2 cells did not differ statistically. Thus, the capacity of *L. intracellularis* to be internalized by other active (self-dependent) mechanisms is very likely.

The main mechanisms involved in bacterial endocytosis are the zipper and the trigger processes. The zipper process is related to the presence of proteins or outer membrane compounds on the bacterium surface that interact directly with host receptors and induce a cascade reaction that culminates with bacterial endocytosis (6, 13). The zipper mechanism has been demonstrated in *Listeria monocytogenes*, which expresses two proteins involved in the internalization mechanism (Internalins A and B—InlA and InlB). Those proteins mimic some host cell proteins and initiate the process of clathrin-mediated endocytosis (8, 14). Although neither InlA, InlB have been identified in *L. intracellularis* genome, as there still are hypothetical and uncharacterized *L. intracellularis* proteins in *L. intracellularis* genome, it is possible that some of them could participate in the bacterial endocytosis. In our study, the presence of clathrin, topographically co-localized with *L. intracellularis* as demonstrated by confocal microscopy, suggests that the *L. intracellularis* internalization process may be clathrin-dependent.

Confocal microscopy has been used to prove the colocalization of bacterial organisms with host cell markers (14–16). In these studies, a semi-quantitative analysis for the confocal microscopy was performed to reinforce the proportion of single positive signals for the bacterial organism, the host cell markers and the double positive signals, allowing more inferences from the data. In the present study the use of confocal microscopy—which specificity is high but lacks sensitivity—was designed to serve as a proof-of-concept for the colocalization of *L. intracellularis* and clathrin, however, a semi-quantitative analysis was not performed. Future studies should consider numerical data to support further colocalization of *L. intracellularis* and host cell markers. Presence of *L. intracellularis* in cytoplasm of IPEC-J2 cells treated with specific siRNA indicates potential internalization by other non-clathrin-dependent mechanisms. To evaluate this, IPEC-J2 cell cultures (control and knockdown for clathrin) were infected with dead *L. intracellularis*. This experiment resulted in a decreased internalization of heat-killed bacteria in cells with diminished clathrin. Based on this experiment results it is plausible to hypothesize that live *L. intracellularis* can be internalized through self-dependent mechanisms, that do not depend on clathrin expression, possibly through the process of endocytosis called trigger. In the trigger endocytosis' process, there is an initial pathogen-host interaction and, from there, bacterial proteins are secreted into the eukaryotic cell cytosol through the T3SS, an apparatus that has already been shown to be present in *L. intracellularis* (17). The secreted bacterial proteins (also called effector proteins) are responsible for host cytoplasmic organization by rearrangements in the actin cytoskeleton (7). This trigger mechanism is well-described in bacteria of the genus Salmonella. Salmonella species adhere to the cells through a fimbrial adhesion and then, through the T3SS-1, synthesize effector proteins responsible for actin polymerization and bacterial envelopment (18). It has been demonstrated (15) that *Salmonella* species can also be internalized by the zipper mechanism mediated by the Rck membrane protein, in addition to the trigger mechanism.

The present study is a proof-of-concept that *L. intracellularis* can be endocytosed by a clathrin-dependent mechanism and/or by mechanisms that require the bacterium's viability. Further studies are needed to determine which outer membrane proteins present in *L. intracellularis* have the ability to interact with host cell receptors to initiate the endocytosis process. Moreover, it is necessary to evaluate whether the T3SS, present in *L. intracellularis*, is involved with the internalization process and to determine the likely effector proteins synthetized by *L. intracellularis*.

## 4. Materials and methods

### 4.1. Cell cultures and *in vitro* infection

Intestinal porcine epithelial cells (IPEC-J2) (19) were cultured in T25 flasks with Dulbecco's Modified Eagle Medium (DMEM)/F12, supplemented with 5% fetal bovine serum, 5 ng/mL epidermal growth factor (E9644, Sigma-Aldrich), and 5 ng/mL mixture of insulin, transferrin and selenium (BD Biosciences), without antibiotics, at 37°C in a humidified atmosphere with 5% CO_2_, as previously described (20). *L. intracellularis* strain PHE/MN1-00 (ATCC PTA-3457), previously isolated from swine with the hemorrhagic form of proliferative enteropathy, was used at passages 12 to 14 to infect the IPEC-J2 cells. A pure culture of the bacterium was thawed and grown in McCoy cell culture incubated in a bag with an atmosphere of ~6% oxygen and 8% carbon dioxide (19, 20), for three consecutive passages to allow bacteria to recover from the freezing condition. Subsequently, the culture supernatant was filtered (0.8 μm, Millipore) to remove McCoy cells which was used as inoculum (21–23), and added into 24-well plates containing IPEC-J2 at ~30% of confluence. As some of the treatment groups were designed to verify the influence of bacterial viability in the outcomes, *L. intracellularis* aliquots were heat-killed by 30 min of heating at 65°C (24). To prove the efficiency of this method, an aliquot of heat-killed *L. intracellularis* was added to a permissive cell culture (McCoy cells at 30% confluence). After monitoring the culture for 6 days, an immunocytochemistry was performed and there were not labeling for *L. intracellularis* antigens (data not shown). As the aim of the immunocytochemistry is to stain *L. intracellularis* in the cytoplasm of the inoculated cells, a negative stain proves that there were no bacteria in the inoculum able to infected and to propagate in the IPEC-J2 cells. Experiments were performed three times, each time with triplicates, with the inoculum containing 2.1 × 10^5^, 3.4 × 10^5^, and 1.8 × 10^5^ *L. intracellularis*/ml and 1.7 × 10^4^, 2.4 × 10^4^, and 2 × 10^4^ IPEC-J2 cells/ml, in each triplicate, respectively.

The experimental groups were: (a) IPEC-J2 knockdown for clathrin (described below) inoculated with viable *L. intracellularis*; (b) IPEC-J2 knockdown for clathrin (described below) inoculated with heat-killed *L. intracellularis* (described above); (c) IPEC-J2 not transfected (control) inoculated with live *L. intracellularis*; and (d) IPEC-J2 not transfected (control) inoculated with heat-killed *L. intracellularis*. The cell cultures were allowed to be in contact with *L. intracellularis* microorganisms for 1 h. After that period, the supernatant was removed, and the cell monolayer was washed three times with PBS and fixed with 4% paraformaldehyde.

### 4.2. RNA silencing

The process of gene silencing through siRNA was performed to evaluate the requirement for clathrin-dependent endocytosis for *L. intracellularis* internalization. The knockdown of the gene clathrin heavy chain like 1 (CLTCL1) responsible for the synthesis of this protein was performed *via* silencing RNA (siRNA) followed by the inoculation of the bacteria (16).

IPEC-J2 cells were transfected using the following oligonucleotide sequences for clathrin synthesis: clathrin heavy chain sense, 5′-GGCCCAGGUGGUAAUCAUUTT-3′; clathrin heavy chain antisense, 3′-AAUGAUUACCACCUGGGCCTG-5 (16). Two days before infection, IPEC-J2 cells were incubated for 15 min with 2.5 μg of siRNA, using lipofectamine 3000 (# L3000008, Invitrogen, USA), according to the manufacturer's instructions. Then, the media was replaced with fresh IPEC-J2 media without the oligonucleotides and the cell culture was infected with *L. intracellularis*.

### 4.3. Western blot

IPEC-J2 cells transfected with siRNA and non-transfected IPEC-J2 cells were washed three times with PBS (25) and lysed with M-PER (#78501, Invitrogen, USA) for 5 min. The lysate was purified by centrifugation at 12,000 g for 10 min. Protein lysates were separated by SDS-PAGE and transferred onto a nitrocellulose membrane and labeled with anti-clathrin antibody (P1663—Cell Signaling, Danvers, MA, USA). β-actin detection was used as a loading control for Western blot experiments with anti-β-actin antibody (C4—sc47778, Santa Cruz Biotechnology). Semi-quantification of protein concentration was performed using imageJ software (National Institute of Health; download from http://rsbweb.nih.gov/ij/).

### 4.4. Confocal microscopy

IPEC-J2 cells were grown on coverslips in 24-well plates and infected with *L. intracellularis* at a multiplicity of infection of 10 at 37°C for 1 h. This period of incubation was chosen after performing a pilot test was performed in which four periods of incubation were tested (15, 30 min, 1 and 2 h) and the highest number of fluorescent signals (*L. intracellularis* and clathrin) was observed at 1 h (data not shown). Subsequently, the cells were washed three times with PBS and fixed in 4% paraformaldehyde for 10 min at room temperature. Thereafter, further washing with PBS was carried out to remove excess paraformaldehyde. Fixed cells were incubated for 30 min at 37°C with the following primary antibodies: anti-clathrin (P1663—Cell Signaling, Danvers, MA, USA; source: rabbit), and anti-*L. intracellularis* (source: mouse) (20). After washing with PBS, the secondary antibodies goat anti-rabbit IgG conjugated to Cy3 (ab97075—Abcam) and goat anti-mouse IgG conjugated to AlexaFluor 488 (ab150113—Abcam) were added and incubated for 30 min at 37°C. Finally, the coverslips were mounted on glass slides with mounting media containing DAPI (ProLong Diamond Antifade Mountant—P36971—ThermoFisher, USA) and imaged under confocal microscope (FV500, Olympus).

### 4.5. Quantification of *L. intracellularis*

To quantify *L. intracellularis* after 1 h of culture, the IPEC-J2 cells were washed three times with PBS to remove extracellular bacteria (25), lysed and processed for intracellular bacterial DNA extraction using DNeasy Blood & Tissue Kit (#D6321-01, Qiagen, Valencia, CA, USA), according to instructions from the manufacturer. The extracted DNA was quantified by real time PCR (RT-PCR) at the University of Minnesota Veterinary Diagnostic Laboratory (UMN/VDL) using standardized methods to estimate the quantity of *L. intracellularis*. The Ct values obtained from the RT-PCR were converted to numbers of bacteria/ml according to an equation previously validated by the UMN/VDL (26). The mean of the results was converted to arbitrary unit (AU) to simplify data presentation, with the results of the positive control group converted to 1 AU and the results of the other treatment group were proportionally converted to the positive control AU.

### 4.6. Statistical analysis

Quantitative data were presented with mean and standard deviation. Statistical analysis was performed using Student's paired *t*-test and considered significant when *P* < 0.05. The software Prism GraphPad version 5.0 was used for statistical analysis.

## 5. Conclusions

In conclusion, the present study has demonstrated the involvement of clathrin in the endocytic process of *L. intracellularis* and has also confirmed that *L. intracellularis* can be internalized through mechanisms that depend on its viability.

## Data availability statement

The datasets presented in this study can be found in online repositories. The names of the repository/repositories and accession number(s) can be found in the article/supplementary material.

## Author contributions

CP, TR, FV, CG, and RG: conceptualization and formal analysis. CP, TR, and AD: methodology. CP and TR: software. CP, TR, and FV: validation. CP, TR, CG, and RG: data curation. CP: writing—original draft preparation. TR, AD, FV, CG, and RG: writing, reviewing, and editing. CG and RG: funding acquisition. All authors have read and agreed to the published version of the manuscript.
